# Transcriptomic analysis of the poultry red mite, *Dermanyssus gallinae*, across all stages of the lifecycle

**DOI:** 10.1186/s12864-021-07547-9

**Published:** 2021-04-07

**Authors:** Kathryn Bartley, Wan Chen, Richard I. Lloyd Mills, Francesca Nunn, Daniel R. G. Price, Stephane Rombauts, Yves Van de Peer, Lise Roy, Alasdair J. Nisbet, Stewart T. G. Burgess

**Affiliations:** 1 Moredun Research Institute, Pentlands Science Park, Bush Loan, Edinburgh, Midlothian, EH26 0PZ UK; 2 Institute of Biological and Environmental Sciences, School of Biological Sciences, University of Aberdeen, Aberdeen, AB24 3FX UK; 3 Natural Resources Institute, University of Greenwich, Chatham, Kent, ME4 4TB UK; 4 Department of Plant Biotechnology and Bioinformatics, Ghent University, Technologiepark 927, 9052 Ghent, Belgium; 5 VIB Center for Plant Systems Biology, Technologiepark 927, 9052 Ghent, Belgium; 6 Bioinformatics Institute Ghent, Ghent University, 9052 Ghent, Belgium; 7 Department of Biochemistry, Genetics and Microbiology, University of Pretoria, Private bag X20, Pretoria, 0028 South Africa; 8 CEFE, CNRS, Univ Montpellier, Univ Paul Valéry Montpellier, EPHE, IRD, Montpellier, France

**Keywords:** *Dermanyssus gallinae*, Poultry red mite, Transcriptome, Lifecycle, Development, Allergen, Blood-feeding, Haematophagous

## Abstract

**Background:**

The blood feeding poultry red mite (PRM), *Dermanyssus gallinae*, causes substantial economic damage to the egg laying industry worldwide, and is a serious welfare concern for laying hens and poultry house workers. In this study we have investigated the temporal gene expression across the 6 stages/sexes (egg, larvae, protonymph and deutonymph, adult male and adult female) of this neglected parasite in order to understand the temporal expression associated with development, parasitic lifestyle, reproduction and allergen expression.

**Results:**

RNA-seq transcript data for the 6 stages were mapped to the PRM genome creating a publicly available gene expression atlas (on the OrcAE platform in conjunction with the PRM genome). Network analysis and clustering of stage-enriched gene expression in PRM resulted in 17 superclusters with stage-specific or multi-stage expression profiles. The 6 stage specific superclusters were clearly demarked from each other and the adult female supercluster contained the most stage specific transcripts (2725), whilst the protonymph supercluster the fewest (165). Fifteen pairwise comparisons performed between the different stages resulted in a total of 6025 Differentially Expressed Genes (DEGs) (*P* > 0.99). These data were evaluated alongside a Venn/Euler analysis of the top 100 most abundant genes in each stage. An expanded set of cuticle proteins and enzymes (chitinase and metallocarboxypeptidases) were identified in larvae and underpin cuticle formation and ecdysis to the protonymph stage. Two mucin/peritrophic-A salivary proteins (DEGAL6771g00070, DEGAL6824g00220) were highly expressed in the blood-feeding stages, indicating peritrophic membrane formation during feeding. Reproduction-associated vitellogenins were the most abundant transcripts in adult females whilst, in adult males, an expanded set of serine and cysteine proteinases and an epididymal protein (DEGAL6668g00010) were highly abundant. Assessment of the expression patterns of putative homologues of 32 allergen groups from house dust mites indicated a bias in their expression towards the non-feeding larval stage of PRM.

**Conclusions:**

This study is the first evaluation of temporal gene expression across all stages of PRM and has provided insight into developmental, feeding, reproduction and survival strategies employed by this mite. The publicly available PRM resource on OrcAE offers a valuable tool for researchers investigating the biology and novel interventions of this parasite.

**Supplementary Information:**

The online version contains supplementary material available at 10.1186/s12864-021-07547-9.

## Background

The poultry red mite, *Dermanyssus gallinae* [22], is the most important haematophagous ectoparasite affecting the global poultry industry. Poultry red mite (PRM) infestations are endemic within poultry farms worldwide, adversely affecting hen welfare and resulting in major economic losses in excess of €231 million per annum in the EU alone [81]. PRM has also been implicated in the transmission of viral and bacterial zoonotic diseases [31]. Many of the traditional acaricides that have been used to control this parasite have either been withdrawn or resistance has developed against them [92]. The recent introduction of a novel acaricide fluralaner, supplied through the drinking water and licensed as a veterinary medicine for use in hens (Exzolt®, MSD) offers the only real current alternative to the traditional sprayer-based control methods [92], however over-reliance on a single class of acaricide is clearly not advisable in terms of development of resistance.

*Dermanyssus gallinae* causes hyperkeratosis and loss of epidermal function in avian hosts [39] and is increasingly being recognised as an important driver of inflammation and allergy in accidental hosts, including humans [13, 31]. The human disease caused by PRM, termed gamasoidosis or dermanyssosis, is characterised by an itchy erythematous maculopapular rash. It is often attributed to PRM from wild birds such as pigeons nesting in urban areas [63] or from back yard hens [66]. The recognition and reporting of gamasoidosis caused by PRM is increasing in urban areas of Europe (e.g. [1, 34]), however human cases in workers within the commercial poultry sector often go unreported. A survey of Italian poultry workers indicate that approximately 20% of workers experience pruritic dermatitis attributed to contact with PRM and suggested PRM should be recognised as an occupational hazard in the poultry sector [14]. The immune response of affected individuals is unknown but it is assumed that, as with other parasitic and free-living mites, PRM may also possess potent allergens.

**Fig. 1:**
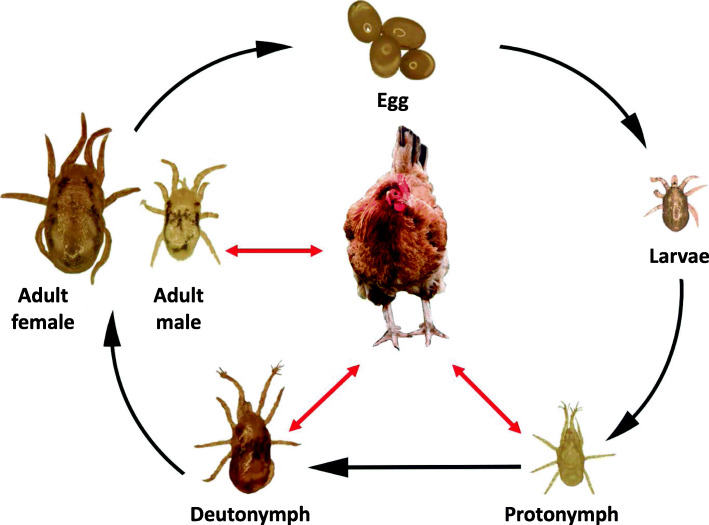
The lifecycle of *Dermanyssus gallinae.* Photographic images of the life stages at 1 week starvation are presented. The red arrows (to and from the host hen) indicate the parasitic stages that are obligate blood-feeders

Research to inform novel methods of control requires detailed knowledge of the unique biology and ectoparasitic life cycle of PRM, which is not fully understood [21]. Poultry red mites spend the majority of their life free-living in the environment and sheltering in the cracks and crevices near to its avian host, only seeking a host when requiring a blood meal. Under ideal conditions PRM can complete its lifecycle (Fig. 1) within 8 days [52]. They are rapid feeders, usually engorging within a 30–60 min time period [53]. Once replete, an adult female mite will produce 1 to 9 eggs, which under optimal environmental conditions will hatch within 24 h to 28 h [82] into the transient hexapod non-feeding larval stage. The larvae undergo a spontaneous moult to the octopod protonymph stage. The highly mobile protonymph stage must ingest a blood meal before ecdysis to the deutonyph stage. A second blood meal is required to complete the ecdysis to the reproductive adult stage. An adult female will feed on average every 2–4 days [53] and begin oviposition within 12 h of feeding. It is not clear how successful the adult male is at feeding, the adult male chelicera is adapted into a large spermadactyl structure used for transfer of sperm during mating, which would make the chewing motion of the chelicera cumbersome. Authors have reported that adult male PRM feed only occasionally [15], Nunn et al. [60] reported that the smaller juvenile stages feed with a greater efficiency when co-feeding with adult females. A co-feeding strategy may explain how adult male are able to acquire occasional blood meals.

For PRM the development of novel methods of control is an ongoing process, which has gained momentum since the upsurge of genomics and the recent publication of transcriptomes and the draft genome of *PRM* [11, 76]. To further exploit these new resources in understanding the biology of this parasite, and to identify potential novel targets for intervention, it is essential that we develop an understanding of the temporal nature of gene expression during development and the associated processes in which the genes function. Such an approach is particularly timely as new tools to understand gene function (e.g. gene silencing by RNAi) have recently been developed and optimised in PRM [16, 43, 99] and some limited transcriptomic and bioinformatics studies on differential gene expression in PRM in different physiological states are starting to emerge (e.g. [3, 30, 41]).

Building on our previously announced PRM draft genome [11], here, we describe the transcriptional profile of PRM genes across each of the 6 defined stages/sexes of PRM lifecycle (egg, larvae, protonymph, deutonymph, adult male and adult female) (Fig. 1); providing a comprehensive, publicly-available transcriptional gene atlas for this species. The patterns of transcription are analysed to demonstrate associations between genes to assist in determining their biologically relevant functions. In addition, we have surveyed the PRM genome to identify putative homologues of defined mite allergens and assess their pattern of expression across the different stages.

## Results

### Functional annotation of the PRM predicted transcriptome derived from the draft genome

The final assembly of the *PRM* genome (Accession Number: QVRM00000000.1) contains 7171 contigs with an N50 value of 278,630 bp and an overall genome GC content of 44.6%. The assembled genome size is ~ 959 Mb containing 14,608 predicted protein coding genes, for which BLAST hits against the NCBI non-redundant (nr) database (July 2018) were obtained for 13,840 genes [11]. The genome assembly is significantly larger than many other mite genomes identified to date, for example those of *Tetranychus urticae* (90.8 Mb), *Psoroptes ovis* (63.2 Mb), *Sarcoptes scabiei* (56.3 Mb), *Dermatophagoides farinae* (53.5 Mb), *Varroa destructor* (294.1 Mb) and *Metaseiulus occidentalis* (151.7 Mb) and more similar in size to tick genomes, i.e. *Ixodes scapularis* (2.1Gb) and *Rhipicephalus microplus* (2.2Gb) where an increase in the degree of non-coding DNA as well as an abundance of repeat sequences have been observed [11, 95]. Gene Ontology (GO) analysis, performed in OmicsBox (Version 1.3.11, Biobam, Spain) resulted in the assignment of GO terms for 11,624 genes and further functional annotation of 10,914 genes.

### Interactive web-based presentation of the entire PRM genome and stage gene expression facilitates interrogation of individual genes and their stage-specific expression profiles

The full annotation of the PRM genome has been made publicly available via the Online Resource for Community Annotation of Eukaryotes (OrcAE: https://bioinformatics.psb.ugent.be/orcae/overview/Degal [87]). To maximise the utility of this information for researchers, for each gene we created a gene-specific page, describing the full annotation available for that gene, including information relating to: gene function, GO, Pfam protein domains, protein homologues and significant BLAST hit data, gene structure, coding sequence, protein sequence and, where available, transcript evidence based on associated ESTs/cDNA data. This PRM gene expression atlas also features a fully searchable database of the entire genome assembly as well as incorporating a display of the Illumina gene expression data across the PRM 6 stages (egg, larvae, protonymph, deutonymph, adult male and adult female) as described here. Each gene has been assigned a unique locus identifier with the following format: DEGALXXgYYYYY, where XX defines the scaffold ID and YYYYY denotes the specific location within the scaffold.

### Expression profiling of genes across the PRM lifecycle

**Fig. 2:**
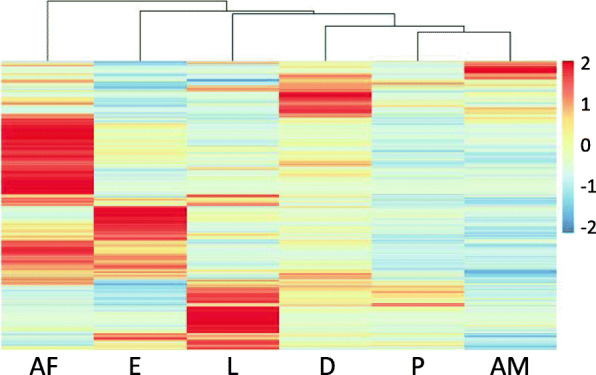
The expression pattern of all *Dermanyssus gallinae* transcripts across the 6 stages. The estimated read count data (transcript per million, TPM) of each transcript in eggs (E), larva (L), protonymphs (P), deutonymphs (D), adult females (AF) and adult males (AM) was mapped using Pheatmap. Red shading indicates high transcript expression, whereas blue shading represents low expression. Only transcripts with an estimated read count value of ≥50 TPM are included

Multiple collections of stage-sorted PRM were pooled and total RNA purified for each stage. Total RNA yields of > 7.5 μg and RNA integrity numbers (RIN values) of greater > 7.2 (range 7.2–9.4) were obtained for each stage. Illumina sequencing resulted in 42–67 million raw sequence reads for each of the six independent sequencing libraries (one for each of the six *D. gallinae* lifecycle/adult sex stages). For each stage a set of expression estimates (transcripts per million, TPM) was generated from the trimmed reads, using the transcript quantification tool Kallisto (Version 0.46.2) [10] and the predicted transcriptome derived from the PRM genome [11], with both sequencing depth and gene length considered in the expression estimate. The expression pattern of all transcripts with a read count of > 50 TPM is presented in Fig. 2 and a clear demarcation of transcript expression between the different stages is apparent. The greatest concentration of highly expressed genes was in the adult females, with some apparent overlap in the expression pattern between adult females and eggs, which could be expected as eggs are also present in the reproductive tract of the adult female.

### Network analysis and clustering of stage-enriched gene expression in PRM

**Fig. 3:**
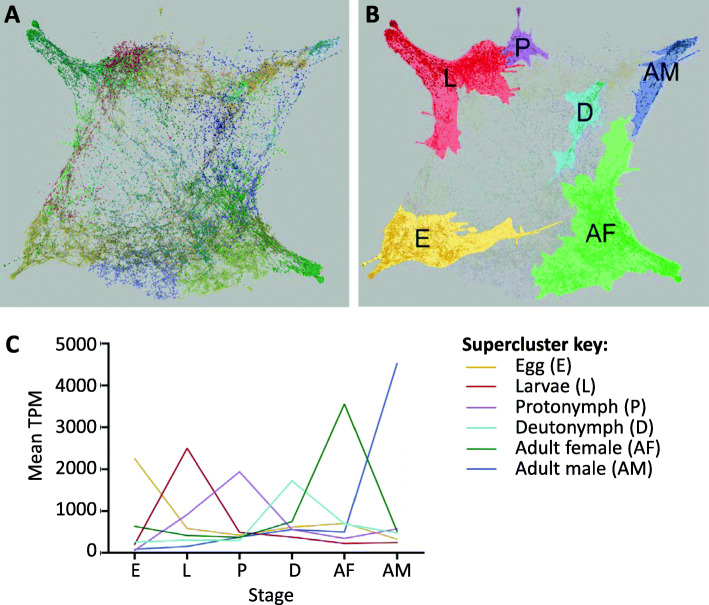
Network analysis of *D. gallinae* transcriptome. Panel **a** shows the entire network graph of the *D. gallinae* transcriptome. Every point (edge) represents one *D. gallinae* transcript and has its own expression pattern across all stages. The location of each point is related to the similarity of their expression pattern. Overall, 44 clusters were identified in the network graph and similar clusters were merged manually to form 17 superclusters. Panel **b** shows 6 superclusters within this network which were highly expressed in single stages where “E”, “L”, “P”, “D” “AM” and “AF” represent transcripts that have high expression in eggs, larvae, protonymphs, deutonymphs, adult males and adult females respectively. Panel **c** shows the mean transcript per million (TPM) data for all *D. gallinae* transcripts in each of the stage-enriched clusters (Superclusters 1–6)

The network analysis of the entire PRM transcriptome (Fig. 3) was performed in order to determine genes expressed in either single or multiple stages of PRM*.* Genes sharing similar signalling pathways and biological functions often display similarities in their patterns of expression and therefore regulation [96] and a similar expression pattern across multiple samples may indicate that they could be involved in similar biological processes, i.e. guilt-by-association [46]. The PRM lifecycle expression network was generated in the Graphia version 2 package [29] using the count data derived from Kallisto. A Pearson correlation cut-off value of ≥0.97 was applied, resulting in a final gene network containing 13,967 nodes (genes) linked by 45,230 edges. Clustering with a Markov Cluster Algorithm (MCL) cut-off of ≥1.2 resulted in the generation of 44 MCL clusters. MCL clusters sharing similar expression patterns across the stages were further merged, resulting in a total of 17 superclusters (Table 1). The distribution of genes across each MCL cluster and supercluster are shown in Supplementary File 1. The genes within each supercluster were mapped back to the original PRM genome annotation and a Gene Ontology (GO) analysis was performed within the Blast2GO/OmicsBox package to identify associated GO terms for molecular function, biological process and cellular component attributed to each supercluster.

**Table 1:** Description of final *D. gallinae* stage-specific gene expression superclusters

Supercluster ID	Predominant expression pattern	Number of genes present in super cluster ^**(Rank)**^
1	Adult Females (AF)	2725^**(1)**^
2	Adult Males (AM)	292^**(13)**^
3	Deutonymphs (D)	295^**(12)**^
4	Protonymphs (P)	165^**(17)**^
5	Larvae (L)	1907^**(3)**^
6	Eggs (E)	1052^**(4)**^
7	E/AF	2480^**(2)**^
8	E/D	178^**(16)**^
9	E/L	743^**(6)**^
10	L/AF	688^**(8)**^
11	L/AM	201^**(15)**^
12	L/D	382^**(11)**^
13	L/D/AF	236^**(14)**^
14	P/D	695^**(7)**^
15	D/AF	899^**(5)**^
16	D/AF/AM	414^**(10)**^
17	D/AM	593^**(9)**^

### Assessment of the most abundantly expressed genes for each PRM stage

**Fig. 4:**
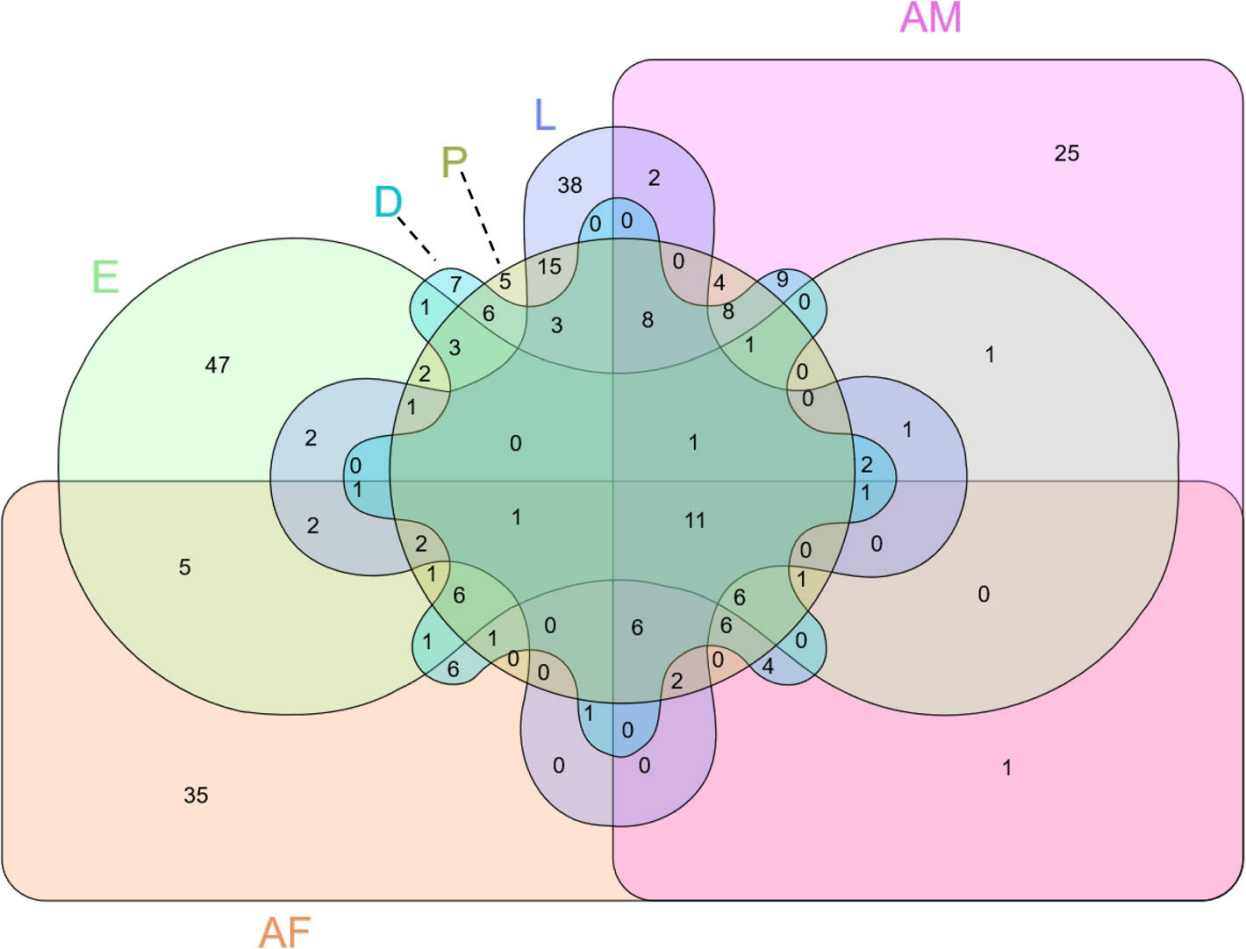
Six-way Venn/Euler diagram of the top 100 most abundant *Dermanyssus gallinae* transcripts of known function for each stage: eggs (E), larvae (L), protonymphs (P), deutonymphs (D), adult females (AF) and adult males (AM). Transcripts were pre-filtered to remove ribosomal proteins and those with no associated annotation, and the top 100 filtered transcripts identifiers for each stage were used in a 6-way Venn/Euler to partition transcript identifiers into unique and overlapping clades

To allow comparison of the most abundantly expressed genes of known function across the PRM lifecycle, we selected the top 100 most highly expressed transcripts from each PRM stage (following removal of transcripts for ribosomal proteins and those with no known function, see Methods section and Supplementary File 2). A six-way Venn/Euler diagram was generated using the top 100 most highly expressed transcripts of known function for each PRM stage (Fig. 4). The transcript identity, associated annotation and expression data (TPM) attributed to each element of the Venn diagram are detailed in Supplementary File 3. The highest numbers of transcripts showing exclusive expression within a specific stage were observed in eggs (*n* = 47) and larvae (*n* = 38); followed by the reproductive adult stages (adult females (*n* = 35) and adult males (*n* = 25)), and finally the feeding juvenile stages, deutonymph (*n* = 7) and protonymph (*n* = 5). To allow comparison of the functions of these highly expressed genes between the individual stages, each transcript was assigned to a broad category indicative of their biological function, which was based on the associated annotation (comprehensive assessment of data from Blastp homology, associated GO annotations and InterPro terms) and is summarised in Supplementary File 4.

### Genes most abundant in multiple life stages and sexes

Examining arms of the Venn diagram with transcripts enriched in more than one stage can be informative for identifying genes associated with stage-specific traits. For example, six genes were present in the Venn sector with highly abundant transcripts present in all blood-feeding stages (protonymphs, deutonymphs and adults) (Fig. 4). These transcripts are therefore likely to underpin the common parasitic biology and processes that are potentially associated with the acquisition, ingestion and digestion of a blood meal. The two most abundant transcripts in the blood feeding stages were DEGAL6771g00070 and DEGAL6824g00220, with estimated TPM values ranging from 36,322 to 71,157. Both of these transcripts are structurally related to each other and have a functional description of “mucin-peritrophin like salivary proteins”. Both proteins are predicted to be glycosylated, DEGAL6771g00070 contains 3 predicted O-linked glycosylation sites and DEGAL6824g00220 contains 7 predicted O-linked and 2 N-linked glycosylation sites. Also, amongst the six transcripts, the protein encoded by DEGAL4040g00020 is a serine endopeptidase belonging to the S1A chymotrypsin family whose members are involved in food digestion, including fibrinolysis [68]. Two serine endopeptidase proteins with multistage expression patterns were also identified (DEGAL4040g00010 and DEGAL2792g 00010) that are structurally (56% identity, E < 3e-108) related to DEGAL4040g00020. Expanded families of genes involved in feeding-associated fibrinolysis are often found in haematophagous arthropods, and are part of the anti-haemostatic pathways essential for keeping ingested blood in a liquid form to allow access for digestive enzymes (reviewed [25, 51]).

### Functional analysis of stage-enriched gene expression in PRM

#### Eggs

There were 1052 transcripts with enriched expression in PRM eggs (Supercluster 6; Table 1) amongst these were a number of genes related to egg hatching and embryonic development including histone, histone-lysine N-methyltransferase and histone deacetylase. In addition, multiple copies of genes involved in cytoskeletal development, translation factors and splicing factors were identified in this supercluster (Supplementary file 1). Analysis of the 47 transcripts exclusive to eggs (termed “E47”) when compared to the top 100 most abundant genes of known function in each stage (Fig. 4 and Supplementary File 4) underlined the abundance of chromatin remodelling proteins involved in histone deacetylation or ATP-dependent histone interaction. Cellular adhesion proteins with known functions in embryogenesis, including gastrulation, were also present in the egg exclusive transcript set.

#### Larvae

We identified 1907 transcripts with enriched expression in PRM larvae (Supercluster 5; Table 1). Of all the mobile stages, larvae contained the highest numbers of genes involved in maintaining the structural integrity of the cuticle (5% of total larval-enriched transcripts). Many of these genes were cuticular proteins (CPs) (10.9, 63, 65, 14 and 14a) which combine with chitin filaments to form flexible or rigid matrixes [62]. The chitin in arthropod larval cuticles is generally translucent and relatively flexible during this stage and is the base for polymerisation and formation of a ridged sclerotized layer in later developmental stages [57]. This sclerotized layer protects the mites from desiccation and mechanical stress, and provides a substrate for muscle attachment [28, 35]. Genes encoding putative allergens, including venom allergen 5, a homologue of the *Lepidoglyphus destructor* mite allergen 7 like and a house dust mite, *Dermatophagoides farinae* allergen group 27 like serpin [2] were also enriched in this stage. Initial formation of the peritrophic membrane in preparation for a blood meal is evidenced by a putative peritrophic membrane chitin binding protein largely found in peritrophic matrixes, which contains the chitin binding protein domains IPR002557 and IPR0365508.

Analysis of the top 100 most highly expressed genes in each stage (Fig. 4) showed 38 transcripts in larvae, which were not present in the top 100 expressed genes of known function for the other stages (termed “L38” transcripts below) and that the largest functional categories of transcripts in larvae were energy metabolism and cuticle proteins with 6 transcripts in each (Supplementary File 4). The energy metabolism transcripts (mitochondrial ATP synthase and cytochrome c subunits) are all involved in the pathway for the synthesis of ATP and none of these transcripts are truly specific to larvae, still having a high abundance in other stages (though approximately 1–5-fold less in other stages). The expanded category of cuticle proteins is, however, specific to larvae: The transcript DEGAL1578g00100, which is present in the L38 transcripts has 76% identity to the tick (*Ixodes scapularis*) RIM-36 cement and cuticle-79 proteins (E < 9e^− 40^) and a further cuticular protein, represented by the L38 transcript DEGAL2920g00060, has an extended RR1 domain, which is a non-cysteine chitin binding domain (non-cysCBD) typically found in the flexible cuticles of larval/pupal stages of arthropods [70] and in the soft endocuticle of other stages [97]. Within the L38 transcripts, there is also a group encoding chitin-binding proteins that are non-cuticular e.g. chitinases, which peak in activity during arthropod ecdysis [102], lectins and peritrophic membrane proteins, which characteristically contain a cysteine chitin binding domain (cysCBD).

Within the L38 group, transcripts DEGAL3518g00030 and DEGAL6700g00030 encode a 168aa glycine-rich Ctenidin-like protein that has been shown to have antimicrobial properties, specifically against gram-positive bacteria [5].

#### Protonymphs

Supercluster 4 contained 165 transcripts specifically enriched in the protonymph stage, and was the smallest supercluster amongst the six stages. During the protonymph stage, mites become more mobile and actively seek out and acquire their first blood meal, which is required for further development [67]. This increase in activity is reflected by a wider range of receptors sensitive to external stimuli and genes involved in the preparation for, and digestion of, blood meals. Within this cluster, 5 genes were identified belonging to the iGluR gene superfamily. This gene superfamily is ubiquitous amongst arthropods [20, 74, 98] and is likely to be the primary modality of olfaction in mite species [26, 33]. Analysis of the top 100 most highly expressed genes in each stage (Fig. 4) showed that a limited number of transcripts, 5 and 7, were exclusive to protonymph and deutonymph Venn clades, respectively; indicating that there are relatively few highly abundant transcripts that have a protonymph or deutonymph specific expression pattern. The TPM values of the transcripts in these two nymph stages all showed multi-stage expression profiles (see Supplementary File 3).

#### Deutonymphs

Network clustering analysis identified 295 transcripts (Supercluster 3) with deutonymph enriched expression patterns. The expression of genes involved in ATP-binding activity is higher in the deutonymph stage than all other stages, with ~ 16% of the deutonymph stage enriched genes in the network clustering analysis involved in this process. As the deutonymphs were not sex-sorted in this analysis, some transcripts, which were later demonstrated to be enriched in different sexes in adult PRM are also present in this final pre-adult stage. For example, cathepsin L2 (CatL; *n* = 2), insulin degrading enzyme (n = 2), serine protease (*n* = 1), serine/threonine-protein kinase (*n* = 27) are enriched here, but are also expressed in adult females. Transcripts involved in muscle and dorsal formation (e.g. 3 copies of dishevelled-associated activator of morphogenesis 1), cuticle development related genes (*n* = 7), and venom allergens (*n* = 3) are enriched here, but are also expressed in adult males. In addition, this supercluster also contained 9 copies of a highly expressed gene encoding the functionally uncharacterised protein BIW11 with an average read count over 250 in deutonymphs. Analysis of the 7 deutonymph exclusive genes in the top 100 most highly expressed genes in each stage (Fig. 4), termed “D7” transcripts here, identified a D7 transcript encoding a calnexin homologue (DEGAL6897g00080), which stores and holds calcium in the endoplasmic reticulum and binds and retains incompletely folded N-glycosylated proteins whilst protein maturation occurs, thus preventing premature destruction of unfolded proteins [49]. Another D7 transcript (DEGAL5401g00010) encodes a homologue of a perlwapin-like mollusc protein that prevents calcium crystallization [94]. It is unclear what the function of this protein may be in a non-mollusc species, but it is interesting to note that the blood calcium levels of adult laying hens is approximately 3-fold higher than in mammals [42] and this protein may assist in preventing calcium crystallization in the gut, haemolymph or biomineralisation of the cuticle.

#### Adult females

Network clustering analysis revealed a total of 2725 transcripts with adult female-enriched expression patterns, which is the largest stage specific cluster in this study (supercluster 1; Table 1). Genes encoding proteins with roles in oogenesis (vitellogenin 1 (*n* = 2), vitellogenin 2 (*n* = 6), vitellogenin receptor (*n* = 3), and apolipophorins (*n* = 3)) were highly expressed in the adult females. Additional reproduction-related genes were also identified in this supercluster including Beta-1,4-mannosyltransferase/Egh, which is a key component of the oocyte-follicle cell adhesive system; chorion peroxidase; beta-1,3-galactosyltransferase/Brn; peroxidase-like isoform X2 and 3 copies of peroxidase-like isoform X3. Other transcripts represented in this supercluster included: heat shock proteins (HSPs), HSP-binding proteins and antioxidants (e.g. peroxiredoxin 1, glutathione reductase, DNA repair factor IIH helicase subunit XPD, thioredoxin-2 and the hypoxia response element, delta-aminolevulinic acid dehydratase). As one of the feeding stages in PRM, several blood meal digestion and metabolism related transcripts were enriched in the adult females, including two copies each of the proteases cathepsin D and cathepsin L (CatD and CatL). Haem released from the digestion of haemoglobin can be toxic to blood-feeding organisms and transcripts encoding proteins putatively involved in haem-handling in adult females included allene oxide synthase-lipoxygenase protein, 4 peroxidases (2 isoform X2 and 2 isoform X3), 2 cytochrome C, 7 Cytochrome P450, sulphite oxidase and chorion peroxidase (reviewed [101]). In addition, the insulin-receptor signalling pathway was also highly represented in this supercluster by insulin-degradation enzyme, insulin-like growth factor-binding protein, insulin receptor substrate 1 and large subunit GTPase 1.

Venn analysis of the top 100 most highly expressed genes in each stage (Fig. 4) showed that 35 transcripts partitioned in the adult females clade (termed “AF35” transcripts below). The most abundant transcripts in the AF35, with the highest TPM values, represented the vitellogenins (DEGAL5400g00090 and DEGAL3689g00030) and a vitellogenin receptor (DEGAL2803g00030) that are uniquely associated with yolk lipid transport and uptake in the developing oocyst. The largest functional category amongst the AF35 contained 12 transcripts encoding proteins associated with nucleic acid binding, predominantly histones and helicases, one of which (DEGAL1221g00050) was associated with the GO term “gamete formation”. The remaining 5 nucleic acid binding proteins have more diverse nucleic-acid binding descriptions (“Other function”) including: tRNA-splicing ligase, chromatin structure regulation, Argonaute gene silencing, RNA decapping and a Zinc finger transcription factor. Other AF35 proteins likely to be involved in cellular expansion are the 3 alpha-tubulin transcripts (Cytoskeleton category) that are associated with cytoskeleton organisation of the mitotic spindle [56].

The second largest AF35 category contained transcripts associated with arthropod innate defence mechanisms, including those potentially involved in mitigating oxidative stress: HSP70 (DEGAL4639g00020, DEGAL3163g00010, DEGAL6541g00010) and a peroxiredoxin, DEGAL4937g00010) and one potential complement binding protein (DEGAL3914g00030).

#### Adult males

Gene ontology analysis of the 292 genes enriched in the adult male supercluster revealed that 43% of these genes were related to metabolic processes (supercluster 2; Table 1). Hydrolases including serine proteases (*n* = 39) and cysteine proteases (*n* = 13), were highly represented in the adult males supercluster. Many of these hydrolases are also present in the predicted secretome of PRM [75] and have also been identified as potential allergens [72].

Analysis of the top 100 most highly expressed genes in each stage (Fig. 4) showed 25 transcripts in adult males, which were not present in the top 100 for other stages (termed “AM25” transcripts below). Proteolytic enzymes comprise the largest functional category in the AM25, including 6 cysteine-type peptidases and 5 serine endopeptidases. In addition, two transcripts encoding serpins were identified in the AM25 set (DEGAL5529g00010, DEGAL6577g00030) both with the domains associated with Kunitz-type serine protease inhibitors. The most abundant transcript in the AM25 was DEGAL6668g00010 which has a > 42-fold increase in relative expression over any other stage. It encodes a Niemann-Pick C2 epididymal secretory protein, which is similar in domain structure to the group 2-like allergens, however, unlike the other group 2 allergens identified in this study (see Allergens section, below), DEGAL6668g00010 lacks a significant homology with the house dust mite (HDM) protein group 2 allergen (E = 0.037).

A chitin-binding protein (DEGAL3530g00010) normally associated with peritrophic membrane/matrix was also identified in the AM25 set. Although the transcript for this protein was identified in all blood feeding stages, in adult males its relative expression was 3-fold higher than any other stage.

### Genes differentially expressed between PRM stages

In total, 15 pairwise comparisons were performed at two simulation probability cut-offs (*P* > 0.95 and *P* > 0.99) between the different PRM stages as shown in Table 2, resulting in a total of 10,122 (*P* > 0.95) or 6025 (*P* > 0.99) genes that were identified as being significantly differentially expressed in at least one of the selected pair-wise comparisons. The list of all DEGs and their log2 ratio (M value) are displayed in Supplementary Files 5 (*P* > 0.95) and 6 (*P* > 0.99). Here we have focussed on the most biologically relevant transitions or comparisons between stages and sexes, namely: adult females (AF) vs adult males (AM); deutonymphs (D) vs adult females (AF) or adult males (AM); larvae (L) vs protonymphs (P) and eggs (E) vs adult females (AF) at the simulation probability cut-off of > 0.99.

**Table 2:** Numbers of differentially expressed genes (DEGs) between *D. gallinae* stages

Comparison^**a**^	Number of DEGs, simulation probability > 0.95^**b**^	Number of DEGs, simulation probability > 0.99^**b**^
E vs. L	5619 (↑2697 ↓2922)	2517 (↑814 ↓1703)
E vs. P	5123 (↑2626 ↓2497)	2270 (↑877 ↓1393)
E vs. D	4791 (↑2204 ↓2587)	1824 (↑605 ↓1219)
E vs. AM	4888 (↑2229 ↓2659)	1882 (↑629 ↓1253)
E vs. AF	4654 (↑1886 ↓2768)	1929 (↑628 ↓1301)
L vs. P	3743 (↑1961 ↓1782)	1352 (↑776 ↓576)
L vs. D	5118 (↑2529 ↓2589)	2306 (↑1190 ↓1116)
L vs. AM	5177 (↑2544 ↓2633)	1951 (↑863 ↓1088)
L vs. AF	5729 (↑2692 ↓3037)	2422 (↑1157 ↓1265)
P vs. D	3063 (↑1545 ↓1518)	720 (↑258 ↓462)
P vs. AM	4398 (↑2087 ↓2311)	1117 (↑361 ↓756)
P vs. AF	5086 (↑2373 ↓2713)	1554 (↑590 ↓964)
AM vs. D	3457 (↑2849 ↓1608)	873 (↑606 ↓267)
AF vs. D	4338 (↑2248 ↓2090)	1326 (↑818 ↓508)
AF vs. AM	4624 (↑2261 ↓2363)	1625 (↑771 ↓854)

^a^*AF* adult females, *AM* adult males, *D* deutonymphs, *P* protonymphs, *L* larvae, *E* eggs. Direction of fold change is relative to the second stage, or condition for each comparison; for example, E vs L, where L is the Reference and E is the Comparison

^b^The numbers of DEG is given in black. In brackets, the red arrows indicate the number of DEG upregulated and the blue arrow those that are downregulated). DEGs were determined using a probability cut-off value of 0.95 and 0.99

### Adult females (AF) vs. adult males (AM)

Overall, there were 1625 genes differentially expressed between AF and AM, and 771 of these were upregulated in AM, whilst 854 were upregulated in AF. Genes with the highest differential expression in AF compared to AM, encoded vitellogenins (DEGAL5400g00090, DEGAL3689g00030); tensin-like proteins (DEGAL2625g00040 and DEGAL2625g00020) and histone-associated transcripts. Two serine protease-encoding genes (DEGAL5835g00120, DEGAL1643g00030) were highly expressed in AF with up to 130-fold change compared with AM. The three genes with the highest differential expression in AM compared with AF encoded a CatL-like protein (DEGAL5953g00010); a legumain-like protease (DEGAL4163g00020), and hypothetical protein BIW11_05264 (DEGAL6170g00010). Overall, proteolysis-related genes were upregulated in AM compared with AF, including 11 transcripts encoding legumain, 24 transcripts encoding CatL, 4 transcripts encoding CatD, and 7 transcripts encoding chymotrypsin-like proteins. Transcripts encoding allergens (see below) were also enriched in AM compared with AF.

### Adult females (AF) vs. deutonymphs (D)

The comparison of adult females and deutonymph gene expression can indicate changes involved in sexual maturation from deutonymph to the ovigerous adult female stage. In total, 1326 genes were differentially expressed between these two stages, and 818 genes were upregulated in adult females, while 508 were downregulated with respect to deutonymphs. The top 3 most upregulated genes in AF encoded vitellogenins 1 (DEGAL5400g00090, 35,929-fold higher expression in AF) and 2 (DEGAL3689g00030, 84,343-fold higher expression in AF) and tensin-like isoform 1 protein (DEGAL2625g00040, 24,080-fold change). The top 3 most upregulated genes in deutonymphs encoded homologues of a kelch-like protein 10 (DEGAL5866g00040), which functions in protein binding, CRE-DIG-1 protein (DEGAL5234g00050), and an uncharacterized protein (DEGAL5253g00030), all with ≥1940-fold higher expression in deutonymphs compared with adult females. A further group of 15 ATP-related genes were highly expressed in AF compared to deutonymphs, consisting of ATP-dependent RNA helicase (*n* = 5), ATP-binding cassette (*n* = 3), ATPase (*n* = 3), DNA replication ATP-dependent helicase (*n* = 1), ADP/ATP translocase (*n* = 1), Werner syndrome ATP-dependent helicase (*n* = 1) and ATP carrier protein (*n* = 1). Another group of 30 histone-related genes were upregulated in AF, including histone-lysine N-methyltransferase (*n* = 13), H2A (*n* = 4), H2B (*n* = 1), H3 (n = 3), H4 (*n* = 1), histone acetyltransferase (*n* = 2), histone chaperone (*n* = 1), set1/Ash2 histone methyltransferase complex (*n* = 1), histone RNA hairpin-binding protein (*n* = 1), histone acetyltransferase (*n* = 2), histone deacetylase (*n* = 1), indicating the potential chromatin regulation and DNA strand compacting during the oogenesis and cellular development of developing larvae contained within the reproductive tract of the adult female. Of 3 transcripts encoding for arginine kinase, two were upregulated in AF, while the third showed higher expression in deutonymphs, indicating that there might be different isoforms or families of arginine kinase regulating phosphotransferase activity in different stages. A further 4 transcripts encoding serine proteinases were upregulated in AF, with up to 122-fold change. Transcripts upregulated in deutonymphs included those encoding a calcium ion binding protein, peflin (*n* = 4) and a kelch-like protein (*n* = 4) functioning in ubiquitination and protein binding.

### Adult males (AM) vs. deutonymphs (D)

This pair-wise comparison provides information relating to male maturation from the final nymph stage and identified 873 differentially expressed genes of which 606 were upregulated in AM, while 267 were downregulated in deutonymphs. Among AM upregulated genes, the top 3 most differentially expressed genes encoded for homologues of an uncharacterized protein LOC111253214 (DEGAL5539g00020, 2296-fold higher in AM), cuticle protein 10.9 (DEGAL6018g00220, 1495-fold higher in AM), and hydrolase activity related pancreatic lipase-related protein 2 (DEGAL7063g00020, 254-fold higher in AM). An additional group of 10 cuticle formation related genes, including cuticle protein 7 (*n* = 4) and 10.9 (*n* = 6) were upregulated in adult males compared with deutonymphs, as were 18 genes representing serine carboxypeptidases and 12 genes representing legumain, which were all highly expressed in AM with up to 188-fold increased expression.

Among the deutonymph upregulated genes, the top 3 highly differentially expressed genes represent homologues of a centrosomal protein of 97 kDa-like isoform X1, which functions in protein binding (DEGAL2866g00020, 99-fold higher in deutonymphs); an organic cation transporter protein (DEGAL3613g00020, 8-fold higher in deutonymphs) and a peptidase activity-related protein: chymotrypsin elastase family member 3B (DEGAL3923g00040, 8-fold higher in deutonymphs).

### Larvae (L) vs. protonymphs (P)

This stage transition represents the transition from free-living, non-feeding larvae to parasitic protonymphs and we identified 1352 differentially expressed genes between these stages. Of these, 776 genes were upregulated in larvae with 576 downregulated with respect to the protonymphs. Of the 776 upregulated genes in larvae, the top 3 differentially expressed genes encoded a homologue of a cuticle protein (DEGAL5073g00020, 18,982-fold higher in larvae), endochitinase-like isoform X1 (DEGAL1215g00020, 12,114-fold higher in larvae) and cuticle protein 63 (DEGAL5246g00010, 11,843-fold higher in larvae). A group of 86 genes encoding homologues of cuticle proteins were highly upregulated in larvae. For protonymph upregulated genes, the 3 most differentially expressed genes encoded two homologues of phosphatidylinositol phosphatase (DEGAL1303g00030, 1039-fold higher in protonymphs and DEGAL1303g00050, 700-fold higher in protonymphs) and a cuticle protein (DEGAL3159g00010, 505-fold higher in protonymphs).

### Eggs (E) vs. adult females (AF)

The comparison of gene expression between eggs and adult females is important to clarify which genes are expressed in the tissues of the adult female mite rather than in the eggs, which are contained within. Of a total 1029 differentially expressed genes, 628 genes were upregulated in eggs with 1301 genes downregulated with respect to adult females. Of the genes upregulated in the eggs, the top 3 most differentially expressed genes all encoded homologues of uncharacterised proteins; LOC100900955 (DEGAL5376g00010, 57,726-fold higher in eggs) and protein BIW11_01270 (DEGAL4071g00020 and DEGAL3196g00010 with 16,607- and 11,909-fold higher expression in eggs, respectively). Of the 1301 genes upregulated in adult females, the top 3 differentially expressed genes encoded homologues of vitellogenin 2 (DEGAL3689g00030, 81,556-fold higher in AF), an uncharacterised protein (DEGAL4206g00020, 26,617-fold higher in AF), and vitellogenin 1 (DEGAL5400g00090, 17,360-fold higher in AF). Overall, there were 10 vitellogenin-related genes upregulated in AF compared with eggs; vitellogenin 1 (*n* = 2), vitellogenin 2 (*n* = 3), and vitellogenin receptor (*n* = 5). Histones and histone-related genes were also upregulated in both egg and AF with different fold change levels. For example, the following genes were identified as being upregulated in AF: histone H1 (*n* = 2), H2A (*n* = 1), H2B (*n* = 4), H1/H5 (*n* = 1), H3.3 (*n* = 2), histone-binding protein Caf1 (*n* = 1), histone demethylation protein (*n* = 1), histone-lysine N-methyltransferase (*n* = 5), histone demethylase (*n* = 2), histone deacetylase (*n* = 1); whereas H3 (*n* = 2), histone-lysine N-methyltransferase (*n* = 13), histone acetyltransferase (*n* = 1), histone deacetylase (*n* = 1) were upregulated in eggs.

### Dermanyssus gallinae putative allergens

**Fig. 5:**
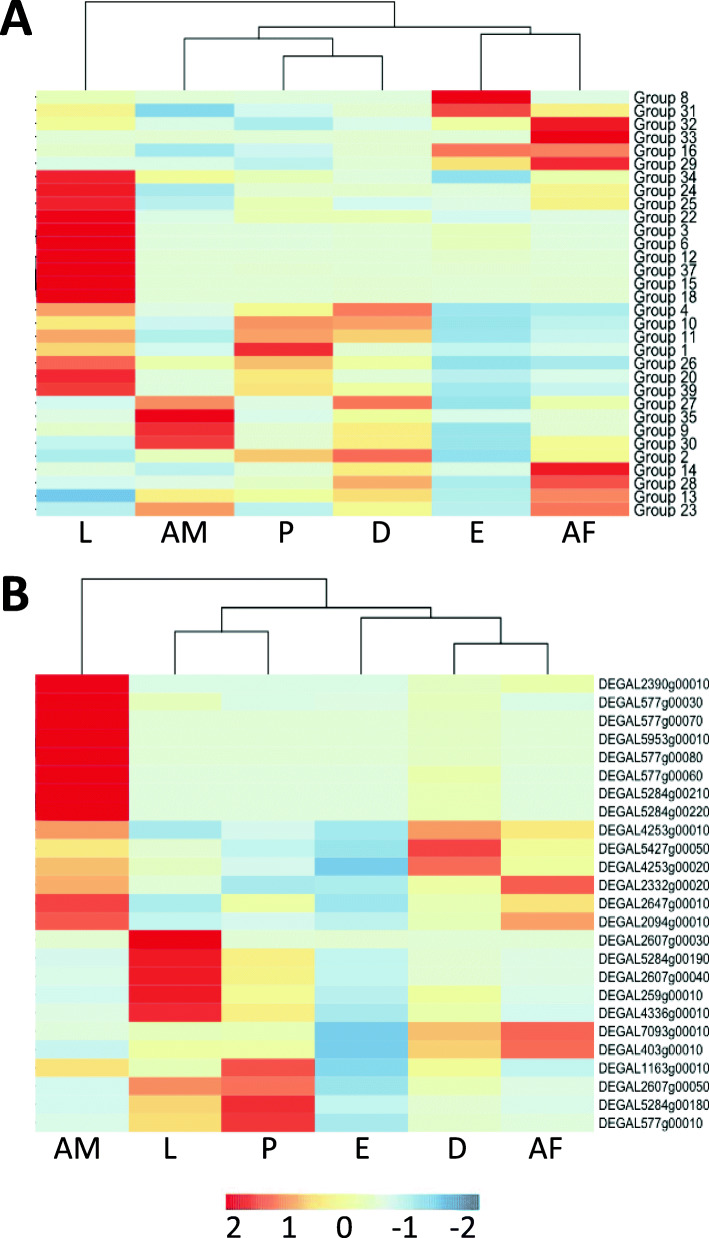
Heatmaps depicting the expression profiles of the *Dermanyssus gallinae* homologues of house dust mite allergen groups across the 6 stages: eggs (E), larvae (L), protonymphs (P), deutonymphs (D), adult females (AF) and adult males (AM). The darker red shading indicates a higher transcript per million value (TPM). Panel **a** depicts the expression level of the top Blastp hit for each of the 39 house dust mite allergen groups classified by the WHO/International Union of Immunological Societies Allergen Nomenclature Subcommittee. No allergen homologues were detected for groups 5, 7, 17, 19, 21, 36, and 38. Panel **b** depicts the expression (TPM, transcripts per million) of the 25 *Dermanyssus gallinae* homologues of the *Dermataphagoides farinae* group 1 allergen Der f 1

Currently, a total of 39 allergen groups have been classified for house dust mites (HDM) by the WHO/International Union of Immunological Societies Allergen Nomenclature Subcommittee (WHO/IUISAN http://www.allergen.org/) based on their predicted immunoreactivity and function. BLASTp homology searching (with a cut off of E < 10^− 05^) of the inferred proteome of PRM with selected archetypal mite allergens from the HDMs, *Dermatophagoides pteronyssinu*s, *D. farinae* and *Bloomia tropicalis* and the Astigmatid mite *Psoroptes ovis* identified homologous PRM proteins. Confirmation of the expression of these genes was acquired by analysis of associated transcript data for all homologues. In addition, conservation of functional domains and active sites for the major allergen groups 1 and 2 were confirmed by bioinformatics analysis. Transcripts lacking these conserved functional elements (e.g. non-functional homologues and pseudogenes) were discarded. Using these selection criteria, homologous proteins belonging to 32 of the 39 defined allergen groups were identified in the predicted PRM proteome. The PRM genes representing these putative allergens with the top BLASTp hit against the allergens from the other mite species, and which met the inclusion criteria for each allergen group (as described above) are described in Table 3. No PRM protein homologues were identified for the allergen groups: 5, 7, 19, 21, 36 and 38. In addition, a BLASTp search for Group 17 allergens could not be performed as the sequence was not available in the WHO/IUISAN database or literature. The expression profiles of the genes encoding the top BLASTp hits for the 32 allergen groups encompassed all stages of PRM (Fig. 5). The larval life stage had the highest expression levels of the genes encoding the highest numbers of allergen groups (14 allergen groups). The lowest expression of top BLASTp hits to the allergen groups was seen in the protonymph and deutonymph stages.

**Table 3:** The top Blastp hit of *Dermanyssus gallinae* proteins homologous with house dust mite allergen groups

Group	Function	BLASTp query	Top hit	Gene Description	Score	E value
1	Cysteine Protease	Der f 1 BAC53948	DEGAL5284g00180	Ctss protein cathepsin S protein cathepsin S	132	1.00E-31
2	MD-2 ML-lipid recognition/NPC2	Der p 2 AAF86462.1 PSOVI17g08010	DEGAL3867g00080 DEGAL6668g00010	Der f 2 allergen/epididymal secretory protein Der p 2-like allergen	65.5 42.7	5.00E-12 3.00E-05
3	Serine protease - trypsin-like	Der p 3 AAA19973.1	DEGAL1643g00010	Brain-specific serine protease 4 stubble-like	159	8.00E-40
4	Alpha amylase	Der f 4 ABG35122.1	DEGAL4003g00110	Alpha-amylase-related protein	62	4.00E-10
6	Serine protease - chymotrypsin-like	Der f 6 AAF28423.1	DEGAL1643g00010	Brain-specific serine protease 4 stubble-like	107	4.00E-24
8	Glutathione-S-transferase	Der p 8 AAB32224.1	DEGAL2189g00680	Gluthathione S-transferase-3 mu	215	7.00E-57
9	Serine protease - collagenase-trypsin-like	Der p 9 AAP57077.1	DEGAL3923g00020	Venom protease, serine protease proclotting enzyme	110	6.00E-25
10	Tropomyosin	Der p 10 CAA75141.1	DEGAL59g00010	Tropomyosin-1, Sar s 10 allergen	310	3.00E-85
11	Paramysosin	Der p 11 AAO73464.1	DEGAL2491g00020	Long form paramyosin	1269	0
12	Chitin-binding/peritrophin-A (chitinase)	PSOVI17g04210 PSOVI14g10800	DEGAL3304g00010 DEGAL1194g00010	Endochitinase chitinase 5 Peritrophic membrane chitin binding protein	53.5 258	1.00E-07 4.00E-69
13	Lipocalin - Fatty Acid Binding Protein	Der p 13 ADK92390.1	DEGAL2772g00010	Fatty acid-binding protein	99.8	2.00E-22
14	Vitellogenin-like	Der p 14 AAM21322.1	DEGAL5202g00200	Vitellogenin-like	46.6	7.00E-05
15	Chitinase	Der p 15 AAY84564.2	DEGAL557g00040	Chitinase 3C	335	2.00E-92
16	Gelsolin	Der f 16 AAM64112.1	DEGAL6121g00060	Flightless-1 protein, gelsolin domain	119	2.00E-27
18	Chitin-binding (chitinase)	Der p 18 AAY84563.1	DEGAL557g00040	Chitinase 3C	196	1.00E-50
20	Arginine kinase	Der p 20 ACD50950.1	DEGAL2192g00450	Creatine-adp Arginine Kinase Ternary Complex	621	1.00E-178
22	Lipid binding	Der f 22 ABG35122.1	DEGAL7041g00020	NPC2-like Protein	68.2	1.00E-12
23	Peritrophin - chitin binding protein	Der p 23 ACB46292.1	DEGAL2104g00010	Acidic mammalian chitinase	43.5	1.00E-05
24	Ubiquinol-cytochrome c reductase binding protein (UQCRB)-like	Der p 24 ALA65345.1	DEGAL4789g00040	Ubiquinol-cytochrome c reductase 14 kDa subunit	68.9	3.00E-13
25	Triosephosphate isomerase	Der p 25 QAT18637.1	DEGAL376g00010	Triosephosphate isomerase	344	1.00E-95
26	Myosin alkali light chain	Der p 26 QAT18638.1	DEGAL877g00070	Myosin light chain alkali	140	7.00E-35
27	Serpin	Der p 27 AIO08851.1	DEGAL4955g00010	Serpin B6-like antithrombin-III precursor	157	6.00E-39
28	Heat shock protein	Der p 28 QAT18639.1	DEGAL3456g00110	Heat shock constitutive protein 70	934	0
29	Cyclophilin	Der p 29 QAT18640.1	DEGAL2838g00020	Peptidyl-prolyl cis-trans isomerase E isoform X2	325	1.00E-89
30	Ferritin	Der p 30 QAT18641.1	DEGAL3420g00020	Ferritin GF1 soma	126	1.00E-30
31	Cofilin	Der p 31 QAT18642.1	DEGAL1595g00470	Cofilin/actin-depolymerizing factor homolog	169	3.00E-43
32	Inorganic pyrophosphatase	Der p 32 QAT18643.1	DEGAL4942g00030	Inorganic pyrophosphatase-like	347	3.00E-96
33	Alpha tubulin 1A	Der p 33 QAT18644.1	DEGAL3563g00110	Alpha 1A-tubulin	578	1.00E-165
34	Rid-like protein enamine/imine deaminase	Der f 34 BAV90601.1	DEGAL6811g00140	RidA family reactive intermediate/imine deaminase	84	1.00E-17
35	Group 2 allergen	Der f 35 BAX34757.1	DEGAL1450g00040	Der f 2/Der p 2/Pso o 2-like mite allergen	63.2	3.00E-11
37	Chitin binding protein	Der f 37 AVD73319.1	DEGAL2715g00020	Peritrophin A	50.4	5.00E-07
39	Troponin C	Der f 39 QBF67841.1	DEGAL4488g00100	Troponin C-like protein Troponin C	256	2.00E-69

A single BLASTp hit, that met the inclusion criteria, was identified for 4 allergen groups: groups 4 (alphamaylase), 14 (vitellogenin), 25 (triphosphate isomerase) and 32 (inorganic pyrophosphatase). Multiple related PRM proteins were identified for 31 allergen groups and several were expanded multigene families and contained 11 or more related proteins including: Groups 3, 6 and 9 (serine proteases), Groups 15 and 18 (chitinases) and Groups 1, 8, 28, 29, 33 and 39 representing the cysteine proteinases, glutathione S-transferase, heat shock protein, cyclophilin, alpha-tubulin 1A and troponin C, respectively. The complete list of related proteins for each allergen that meets the inclusion criteria is presented in Supplementary File 7.

## Discussion

Here, we have presented the first transcriptomic analysis of each stage of PRM*,* assessed global and stage/sex-enriched gene expression and performed pair-wise comparisons of gene expression in the most relevant biological comparisons/transitions. Previous analyses of gene expression in PRM have described the transcriptome of mixed life stages [76], comparative transcriptomes of fed/starved mites [3, 41] and analysis of chemosensory gene expression in different organs [7]. We have also made the transcriptomic data comparisons across stages publicly-accessible for every predicted gene through the OrcAE system.

One of the key transitions in the life history of this mite is the move from a free-living, non-feeding larval stage to a parasitic lifestyle in all later stages of development. A large number of the differentially regulated genes in our analysis relate to this transition. Genes encoding two related proteins: DEGAL6771g00070 and DEGAL6824g00220 were identified in the region of the Venn/Eular analysis representing the blood feeding stage overlap (i.e. protonymphs, deutonymphs and adults). Both of these genes were highly expressed in all blood feeding stages and with the exception of DEGAL6824g00220 in deutonymphs, they were the most abundant transcripts in all blood feeding stages that were represented in the blood-feeding overlap group. The proteins encoded by both transcripts are mucin-peritrophin like salivary proteins containing a characteristic chitin binding peritrophin-A domain (IPR036508/PF01607) and are predicted to be glycosylated, particularly DEGAL6824g00220, which is predicted to be heavily glycosylated, typical of mucin-peritrophin proteins [80]. In some blood-feeding invertebrates, a temporary acellular semipermeable structure, termed the peritrophic matrix/membrane, is formed during feeding. It encloses the ingested blood meal and protects the midgut from enzymatic damage and from pathogens ingested with the blood meal; in addition, acting as a molecular sieve to compartmentalise the gut and aid transport of selected nutrients to the gut surface (reviewed by [38]). Amongst the blood-feeding Acari, the presence and function of the peritrophic matrix has been most closely studied in ticks (e.g. [32]) where it performs important functions in limiting the uptake of tick-borne pathogens [44, 103] but there is a paucity of information on the presence and nature of this structure in PRM to date.

Proteolytic enzymes involved in blood meal digestion also feature heavily amongst the transcripts represented in the later life stages (nymphs and adults). Cathepsin D, legumain, cathepsin L and other digestive cysteine proteinases are all prominent in haematophagous arthropods [85] and have been shown to be integral to the degradation of haemoglobin in ixodid ticks [40]. Cathepsins D and L in particular are thought to initiate the autophagic-lysosomal pathway [58] in PRM and have been identified, and demonstrated, as potential vaccine targets [3, 65].

Reproduction and egg laying are associated with the maturation of the mites and their blood-feeding episodes and the vitellogenin-encoding transcripts (DEGAL5400g00090 and DEGAL3689g00030) and a vitellogenin receptor (DEGAL2803g00030) that are uniquely associated with oogenesis, were highly abundant in adult females. Vitelloginin is a lipid binding protein expressed in the fat body, mid-gut and ovary of fed adult female ticks [9] and the vitellogenin receptor mediates the active uptake of vitellogenin into developing oocysts [8].

The abundant adult male transcript DEGAL6668g00010 (> 42-fold increase compared to other stages) encodes a protein, which possesses InterPro domains associated with cholesterol binding Niemann-Pick C2 proteins (NP-C2). These domains are found in proteins in epididymal secretions of mammals and group 2 allergens. Investigation into the function of this epididymal protein in the crustacean *Penaeus monodon* suggest that it participates in sperm maturation by aiding lipid membrane remodelling [17]. The 3-fold increase in expression of a transcript encoding a peritrophic matrix-like protein (DEGAL3530g00010) in the adult males may indicate function(s) other than those associated with blood feeding. In a number of arthropods, chitin-binding proteins have been identified in the seminal fluid or accessory gland [78]. Proteinases in invertebrates have been traditionally viewed as active salivary or gut enzymes associated with digestion but are now also viewed as having further-reaching roles, with high levels of expression (along with pseudoproteinases) in other tissues associated with reproduction, defence and host immuno-modulation (reviewed by [27]). The proteome of male *Aedes aegypti* accessory glands and ejaculatory ducts show an abundance of proteases (25% of the total proteins identified) and protease inhibitors (11%) [84]. In PRM, we saw a similar enrichment in proteases in the male Venn-clade expression pattern transcripts, with 44% of those identified belonging to the cysteine and serine proteinase families. In addition, arthropod serpins comprise a large part of seminal fluid and their functions are diverse, with evidence suggesting a limited involvement in proteolytic enzyme inhibition, but rather involved in pathogen defence in pathways yet to be elucidated (reviewed by [55]).

A variety of defence-related proteins potentially involved in neutralizing threats arising from infectious agents and the host immune response were particularly evident in the abundance data for adult stages. Adult female PRM feed recurrently and ingest large volumes of host blood (on average every 2 to 4 days and 2.7 times their own body weight) [53, 82] exposing them to a range of these threats, whilst also dealing with the reactive oxygen species (ROS) generated from the digestion of haemoglobin [19]. In relation to this, a transcript encoding a peroxiredoxin protein (DEGAL4937g00010) was enriched in adult female PRM (3.4 to 16.6-fold over other stages) and, in blood-feeding arthropods, peroxiredoxins have multifunctional protective roles and are implicated in neutralizing ROS arising from oxidation of ingested haem and from the host response [50]. HSP70 is associated with antiviral defence in arthropods, possibly by assisting in the loading of siRNA complexes as part of the anti-viral RNA silencing machinery [100]. Several HSP-70-like proteins were identified in the Venn clades for the adult male and female stages. Transcript DEGAL3914g00030 also encodes a protein, which possesses a sushi/complement control domain potentially capable of binding to avian complement factors C3b and C4b [93] with a potential function in sequestering and neutralizing host immune complement as is seen in other haematophagous parasites [77]. Genes encoding three defensin type proteins were identified in the top 100 most abundant genes in adult males. In the tick *Haemaphysalis longicornis*, an exclusive male-pattern expression of defensins has also been identified, predominantly in the accessory gland, and are thought to have a protective anti-microbial function in the reproductive tracts and confer that protection to the female reproductive tract following mating [104].

There has been very little focus on the larval stage of PRM in prior literature and here we present the first transcriptomic profile of this stage. This stage represents a non-feeding rapid transition stage where the biological imperative is on survival and preparation of a new cuticle for the moult to protonymph. Associated with this is a transcriptional focus on energy metabolism and cuticle-related proteins. Also potentially associated with moulting from larvae to protonymph is the abundant expression of three carboxypeptidase A2-like proteins: In the silkworm (*Bombyx mori*) Ote et al. [61] identified carboxypeptidases expressed in the pupal stage in the intra-cuticular space between old cuticle and new developing cuticle during ecdysis and the three metallocarboxypeptidases identified in the larvae transcripts share a > 37% identity (E = 9e^− 59^) with this protein.

*Dermanyssus gallinae* is increasingly being recognised as an important domestic and occupational health issue, with exposure resulting in inflammatory skin reactions in humans and hyperkeratosis and loss of epidermal function in poultry (reviewed by [13]). However, the full repertoire of allergens present in PRM has not been investigated until now. Orthologues of the HDM allergens have been determined for some Astigmatid mites, including the parasitic mite *Psoroptes ovis,* using a bioinformatic approach [12, 36]. Comparison of the allergens of related mite species with different life-styles can be informative, for example the Trombidid mites: *Leptotrombidium delicense* and *Dinothrombium tinctorium*, that parasitise vertebrate and invertebrate hosts respectively. The parasite of vertebrates, *L. delicense*, was predicted (in silico) to possess a larger allergen repertoire (37 allergen groups) than *D. tinctorium* (32 groups) [24]. Twenty three of these allergens were also homologues of the HMD allergens. Due to the clinical importance in human disease, the allergens of HDM are the best characterised and many HDM allergens are conserved in parasitic mite species. Although, HDMs have a non-parasitic lifestyle, they likely derive from a parasitic ancestor [45] and as such it was chosen as the model to begin the process of identifying allergens in PRM. However, the inference of PRM (a Mesostigmatid mite) protein homology with the Astigmatid HDM is more problematic due to greater evolutionary distance and the absence of defined allergens in species more closely related to PRM. Even amongst closely related HDM species (e.g. *Euroglyphus maynei* and *Dermatophagoides* spp.), allergens fall within multigene families and there is a complexity in determining the allergen orthologues [73]. Using an approach combining a cut-off value of E < 10^− 05^, transcript evidence, phylogenetics and functional domain conservation in multigene families, we were able to identify a single protein or a small number (< 8) of related proteins for 21 allergen groups in PRM. A further eleven allergen groups (1, 3, 6, 8, 9, 15, 18, 28, 29, 33, 39, and) had expanded sets of related genes in PRM*,* which belonged to large multigene families: cysteine and serine proteases, GSTs, chitin binding/chitinase proteins, HSPs, cyclophilins, and structural components alpha tubulin 1A and troponin C. This situation was comparable to the expanded allergen group homologues seen in *P. ovis* (groups 8, 9, 15, 27, 28, 29 and 33) and *E. maynei* (groups 1, 3, 6, 9, 15, 18, 27, 28, 29) [12, 73] and is representative of the difficulty in identifying the true orthologous allergen proteins using an in silico approach alone. Identifying the allergen IgE targets of sensitised humans and poultry would be useful in fully comprehending the allergen repertoire of PRM. Surprisingly, the response of hens to normal population sizes of feeding mites (~ 50,000 mites per hen) is limited to hyperkeratosis and damage in the epidermis [13] and there have been no reports of severe allergic responses or anaphylaxis in hens following PRM infestation. Strategies to modulate the host immune response are well described for numerous ectoparasites (e.g. [6, 48]) and generally rely on secretion of immunomodulatory factors to up or down regulate the host’s immune response depending on what the parasite’s survival requires. Indeed, hard ticks both up and down regulate the host response depending upon the phase of feeding, down-regulating the host response during the vulnerable attachment phase and inducing a vascular inflammatory response during rapid engorgement [83]. The lack of an acute allergic response to feeding mites in hens may also be due to active down-regulation of the host response by immunomodulators and potentially the decreased expression of archetypal mite allergens in the blood feeding stages (Fig. 5a).

Group 1 and 2 allergens are the dominant drivers of IgE-mediated allergy in humans against HDM (Der p 1 and Der p 2) [90]. Twenty-five PRM proteins belonging to the cysteine protease family were homologous to the dominant group 1 allergens and were predominantly expressed in the larval and blood feeding stages, but not eggs (Fig. 5b). The bias in expression towards the blood feeding stages is unsurprising as many of these active cysteine proteases are thought to be involved in digestive processes, as they localise to the midgut of other mite species e.g. HDM and *P. ovis* [54, 59, 91] and are likely to have similar digestive function in PRM. The major group 2 allergens (and the related minor allergen groups 7, 22 and 35) are lipid-binding proteins that have a characteristic Ig-like E set domain fold (IPR014756), MD-2-related lipid-recognition (IPR003172) and a Neimann-Pick C2 intracellular cholesterol transporter 2 (NPC2) domain (IPR039670). Two PRM transcripts (DEGAL3867g00080 and DEGAL4453g00010) had significant homology to Der p 2 and possessed the characteristic lipid-binding functional domains. An additional group 2 allergen homologue (DEGAL6668g00010) was identified with homology to the Pso o 2 allergen from *P. ovis*, but not Der p 2, and was the most abundant transcript in the 6-way Venn analysis adult male clade and the 3rd most abundant transcript in adult males overall. Although the function of these allergens in mite biology remains elusive, the ability of Der p 2 to bind lipid and preferentially cholesterol has been confirmed [71]. The putative valine-rich cholesterol binding site identified in group 2 allergens from HDM [71] appears to be partially conserved in all of the PRM Der p 2 homologues, except the male-enriched male DEGAL6668g00010, potentially indicating a different function to the archetypal Der p 2 allergen, possibly in male reproductive biology (as discussed previously).

The mechanism of PRM allergens interacting with their avian and accidental human hosts is largely based on assumption and extrapolation from other ectoparasite species. Allergen transfer could possibly occur by several routes: 1, invasive transfer of salivary allergens into the host during feeding events, and/or; 2, passively by direct epidermal contact, accidental inhalation or ingestion of allergens present in the cuticle or faeces of PRM. To date there has been no investigation looking specifically at the interaction of PRM allergens and hosts, however evidence exists to support a route of exposure for some PRM allergens. For example, a PRM cathepsin L like proteinase, Dg-CatL-1 (DEGAL577g00010), is related to the major group 1 allergen of HDM and *P. ovis* (Supplementary Table 4). This cathepsin is highly immunogenic and is recognised by immunoglobulins from naturally infested hens [3] and was identified in the secretome of PRM [75]. It is upregulated in fed mites [3] and is therefore likely to be involved in food digestion and excreted into the environment in the mite faeces like its *P. ovis* and HDM counterpart [59, 72] or in saliva during feeding. The overwhelming picture emerging in the literature regarding the symptoms of humans afflicted with dermanyssosis is of a pruritic papule, often with a central puncture wound, suggesting feeding mites may be the dominant route of allergen exposure, however urticoid rashes are also seen and may be indicative of contact reactions (reviewed [13]).

## Conclusions

This study provides the first evaluation of temporal gene expression across all of the stages in PRM and has provided insight into developmental, feeding and survival strategies employed by this mite. For example, the developmental transition from the free-living, hexapod non-feeding larval stage to the parasitic, octopod blood-feeding stages is demarked by the high expression of cuticle proteins and enzymes involved in ecdysis (e.g. chitinases and metallocarboxypeptidases) in larvae to those that are involved in blood feeding in the later parasitic stages. The identification of mucin/peritrophic-A like proteins in the feeding stages, provides the first evidence to indicate that PRM produces a peritrophic membrane during blood ingestion. A suite of defensive proteins that are enriched in the reproductive adult stages and larvae were identified and may be important in preventing microbial infection during vulnerable states for example, mating, hatching and ecdysis.

The identification of putative homologues from 32/39 HDM allergen groups, supports recent evidence arguing that PRM poses occupational hazard to sensitized human poultry workers. The bias in temporal expression of the putative allergens away from the parasitic feeding stages may represent a host immune-avoidance strategy.

The combined resources of the annotated draft genome, transcriptome data and a temporal gene expression atlas, made publically available in the interactive OrcAE platform offers an invaluable tool for research. This expanded genomic resource will underpin future studies into mite biology and the discovery of novel interventions for the control of PRM.

## Methods

### PRM collection and lifecycle staging

*Dermanyssus gallinae* mites were obtained directly from perches and walkways in an organic free-range laying facility housing 2000 laying hens in the Scottish Borders region of the UK. Poultry house detritus containing aggregations of mixed stage and sex mites was collected and contained in Corning® 75 cm^2^ U-shape vented cell culture flasks (Merck). A PRM egg harvesting and a larvae and protonymph rearing protocol was developed to ensure clean and synchronised populations of these stages: To obtain eggs, mixed stage and gender mites were allowed to crawl out of poultry house detritus within 2 h of collection and approximately 1 cm^3^ volumes of mites were transferred to 20 ml plastic universals (Sterilin) and sealed with double layers of AeraSeal™ film breathable adhesive membrane (Merck). Mites were incubated at 25 °C/85% relative humidity for 24 h. Freshly deposited eggs were transferred to a 1.5 ml microcentrifuge tube using an artist’s fine paint brush and, for RNA extraction, were immediately snap-frozen in liquid nitrogen. To rear hexapod larvae, freshly deposited eggs were transferred to a 5 ml plastic bijoux (Sterilin), sealed with AeraSeal™ membrane and incubated for a further 48 h at 25 °C/85% relative humidity to allow hatching into the larval stage. In addition, a longer incubation of 120 h allowed both hatching and additional moulting to the octopod protonymph stage. Prior to snap freezing of eggs, larvae and protonymphs, the mites were examined microscopically and any contaminating stages removed.

Deutonymph and adult stages were isolated directly from the collected material that had been incubated at RT for 1 week prior to stage sorting, ensuring the complete digestion of previously ingested blood meals. The characteristic size and morphology of the deutonymph and adult stages following incubation at RT for 1 week was first determined by preserving mites in 100% ethanol and stage sorting using previously described detailed morphology seen under high magnification (200X) [23, 64]. The staging criteria of preserved mites included: 1. Body shape; males have a roughly anterior to posterior lateral idiostomal tapering, whereas females and deutonymphs have roughly parallel lateral surfaces with a broadly rounded posterior. 2. Ventral shield structures: Adult females have a fully developed genitoventral shield (often wrinkled) that is clearly demarcated from the anal shield, whereas the epigynal shield in deutonymphs is unwrinkled and much reduced in length compared to the adult female (and even shorter in protonymphs). In adult males, there is no demarcation between the epigynal (holoventral) and anal shields. 3. Reproductive structures: the adult male has an anteriorly located genital opening on the holoventral shield and an modified chelicerae, which carry and additional organ, the spermadactyl. 4. The body length (idiostomal length, excluding legs) of all mite stages following incubation at RT for 1 week was determined by examination on 1 mm graticule slide and were consistent at: larvae 0.4 mm, protonymphs 0.4 mm, deutonymphs 0.5 to 0.55 mm, adult males 0.6 mm and adult females 0.8 mm.

Mites free from detritus were transferred from the culture flask cap onto a glass Petri dish to allow large numbers of live deutonymph and adult mites to be stage sorted. Stage sorting was performed using size and body shape as the sole criteria to sort live mites. The accuracy of the stage sorting was confirmed by sacrificing a subsample of each stage sorted collection (approximately 10% of each collection) and performing a more detailed morphological examination on ethanol preserved mites (as detailed above). Following stage sorting, mites were immediately snap frozen in liquid nitrogen and stored at -80 °C until required.

A novel method of harvesting freshly deposited PRM eggs and raising larvae and protonymph stages in vitro was developed to ensure synchrony in subsequent stage development and enable large numbers of eggs, larvae and protonymphs to be recovered [60], resulting in a 100% degree of accuracy (as determined by microscopical examination). Detailed microscopical examination of the ventral shield structures and male genital opening of sacrificed subsamples of each stage-sorted collection confirmed a high degree of mean stage sorting accuracy of 92.1, 97.2 and 91.0% for deutonymphs, adult females and adult males, respectively. In total, approximately 50-100 mg of each mite stage/sex was recovered (from pooled multiple collections) for RNA purification, visually this ranged from a packed volume of 70 μl for adult males to approximately 200 μl for adult females and eggs.

### RNA extraction and quality control

Total RNA was purified from stage sorted mites by grinding mites in liquid nitrogen using a mortar and pestle followed by extraction using TRIzol reagent (Invitrogen) adhering to the manufacturer’s guidelines. Contaminating genomic DNA was removed by additional purification through an RNeasy mini column (Qiagen) with on column DNase treatment for 15 mins at RT. Eluted total RNA was stored at -80 °C. RNA was quantified using a Nanodrop and Qubit Fluorometer with a Qubit RNA BR assay kit (Invitrogen) and RNA quality was assessed using a 2100 Bioanalyzer with RNA 6000 Nano reagents (Agilent) following manufacturer’s protocols.

### Library preparation and transcriptome sequencing

TruSeq RNA-seq libraries (Illumina, USA) were prepared from the six PRM stage RNA samples. RNA was enriched for polyA RNA using TruSeq oligo-dT beads prior to cDNA synthesis using random hexamer priming according to the manufacturer’s instructions. Sequencing was performed on the Illumina HiSeq 4000 platform (Ilumina, USA) by the Centre for Genomic Research (CGR) at the University of Liverpool with 2x150bp paired-end, strand-specific sequencing.

### Bioinformatic analysis

Base calls were made using the Illumina CASAVA 1.8 pipeline. Post-sequencing, read quality of raw FASTQ files was checked with FastQC v0.11.7. The CLC Genomics Workbench (Version 12, Qiagen Ltd) was used for adapter, quality, ambiguity, and length trimming. For alignment of the read data, we employed the draft genome assembly for PRM, which is a ∼ 956 Mb genome containing 14,608 predicted protein-coding genes [11]. Pseudo alignment of the read data to the *D. gallinae* genome (Accession ID: QVRM00000000) was performed in Kallisto (Version 0.46.2) [10] generating read count data for each transcript across all RNA-seq samples, which were used as input for the network clustering within the Graphia package (Version 2.0, Kajeka, UK) and for the analysis of differentially expressed genes between PRM stages.

### Functional annotation analysis

The transcriptome sequencing results were loaded into OmicsBox (Version 1.3.11, Biobam, Spain) before applying “Blast”, “InterProScan”, “Blast2GO Mapping”, and “Blast2GO Annotation” functions. Gene ontology (GO) analysis were then generated at ontology level 2, 3 and group within 17 stage-specific superclusters. Three aspects of GO were displayed in the charts consisting of biological processing, cellular component, and molecular function. In addition, functional metabolic pathways were mapped using the Kyoto Encyclopedia of Gene and Genomes (KEGG) pathway database within OmicsBox.

### Interactive web-based presentation of the D. gallinae genome and stage gene expression

The graph visualisation and analysis package, Graphia (Version 2.0, Graphia Technologies Ltd., UK) was used to display the gene network expression graph. TPM data for all predicted PRM genes (*n* = 14,608) across all stages (× 6) was used as the input dataset. The network graph was generated using a Pearson correlation cut-off value of ≥0.97 with k-nearest neighbours (k-NN) of 5 edge reduction applied. The resulting graph was then clustered using a Markov Clustering Algorithm (MCL) cut-off of ≥1.2, and all components with less than 5 edges (genes) were removed. Finally, gene expression MCL clusters sharing similar expression patterns across all 6 life cycle stages were merged manually into superclusters. Glycosylation prediction was performed using NetNGlyc 1.0 and NetOGlyc 4.0 [86]. For each stage enriched supercluster, the mean TPM values were calculated for all 6 stages, and their average expression profile was generated in GraphPad Prism (version 9.0, GraphPad Software, USA).

### Assessment of the most abundantly expressed genes for each D. gallinae stage

The transcripts for each PRM stage were ordered by transcript abundance (i.e. based on the estimated TPM value data from Kallisto). However, over half of the transcripts (range 51–72 for all life stages) in the top 100 most abundant genes were of ribosomal origin or had little or no associated annotation (Supplementary File 2 - Unfiltered). Therefore, transcripts were prefiltered to remove ribosomal genes and those genes with no known function, obtaining more meaningful gene lists of the top 100 most abundant transcripts for each stage (Supplementary File 2 - Filtered). Venn/Euler analysis was performed with the 6 top 100 abundant (pre-filtered) gene identifier datasets using InteractiVenn [37].

### Determination of differentially expressed genes

Pairwise comparison of differentially expressed genes (DEGs) was performed using the NOISeq package (Version 3.11) with the TPM data from Kallisto for all stages. As the RNA-seq data from the PRM stages was generated in singlicate from large pools of mites, we used the non-parametric, NOISeq package (Version 3.11) to estimate replicates for each of the stages prior to the determination of DEGs [88, 89]. Using the NOISeq-sim method we simulated a total of 5 replicates for each stage based on a replicate size of 0.2 and a variability value of 0.02. We applied an initial counts per million (CPM) cut-off, > 10 reads in all stage samples and data was normalised via the trimmed mean of M (the log2 ratio of two comparisons) values (TMM), where D describes the TPM difference for each gene. Genes were then ranked based on the following calculation (−sign(M)*sqrt(M^2 + D^2)) to provide a set of ranked differentially expressed genes. Also, log2 ratio and fold changes were calculated in all genes in each pairwise comparison.

### Identification of putative D. gallinae allergens

PRM homologues of the current 39 house dust mite allergen groups, classified by the WHO/International Union of Immunological Societies Allergen Nomenclature Subcommittee (WHO/IUISAN http://www.allergen.org/) were identified by Blastp searching of the predicted PRM proteins database (DEGAL_PROT) hosted on OrCAE [11] using the BLOSUM62 matrix with a gapped alignment and an expect cut-off value of E < 1.0E^− 05^. The Blastp query sequence for each allergen group was obtained from published *Dermatophagoides pteronyssinus* or *Dermatophagoides farinae* sequences; however, allergen sequences from *Psoroptes ovis* and *Blomia tropicalis* were used as query sequences when a *Dermataphagoides spp*. sequence was unavailable or lacked significant homology with PRM. Homologous sequences from *D. gallina*e were only considered if there was at least one piece of evidence supporting gene expression identified in the associated RNA-seq datasets: mixed stage 454 [4] and PacBio Iso-Seq [11] and the stage Illumina RNA-seq data (presented in this manuscript). Additional selection criteria was applied to the major allergen groups 1 and 2. Group 1 allergen homologues were only considered if they possessed the conserved elements essential for folding and function: catalytic residues (QCHN), active site domains, semi-conserved cysteine and asparagine residues important for di-sulphide folding and glycosylation [18, 79]. Likewise, group 2 homologues were only considered if they possessed the characteristic Ig-like E set domain fold (IPR014756), MD-2-related lipid-recognition (IPR003172), Neimann-Pick C2 intracellular cholesterol transporter 2 (NPC2) domain (IPR039670) and a putative valine-rich cholesterol binding site identified in HDM [71].

Expression patterns of putative allergen homologues across the PRM stages were visualised by plotting the estimated TPM data using pheatmap package (version 1.0.7) [47] in R (version 4.0.2) [69].

## Supplementary Information

**Additional file 1: Supplementary file 1.** Summary of transcripts in the Markov Cluster Algorithm (MCL) network clusters and their supercluster identity designation corresponding to the 6 life stages (egg, larvae, protonymph, deutonymph, adult female and adult male) of *Dermanyssus gallinae*.

**Additional file 2: Supplementary file 2.** Top 100 most abundant transcripts for each life stage of *Dermanyssus gallinae* (egg, larvae, protonymph, deutonymph, adult female and adult male) based upon the transcript per million (TPM) values. Filtered transcripts: Top 100 most abundant transcripts for each life stage that were manually filtered to removed ribosomal RNA subunits and transcripts with little or no associated annotation. Unfiltered transcripts: Top 100 most abundant transcripts for each life stage with no filtering.

**Additional file 3: Supplementary file 3.** Tabular output of a 6-way Venn/Euler diagram. Constructed from the top 100 most abundant transcripts from each life stage (that were pre-filtered to remove ribosomal proteins and those with no associated annotation). The transcripts localising to the Venn clades of the 6 individual life stages and the 39 multi-life stage clades of *Dermanysssus gallinae* are listed. Transcripts were organized in broad categories termed ‘Gene function category’ and ‘Inferred function’ based on a comprehensive assessment of inferred functions from Blastp homology, and associated GO annotations and InterPro terms.

**Additional file 4: Supplementary file 4.** Summary of inferred transcript functions in selected Venn clades, representing the individual life stages and multiple stage comprising of the ‘blood feeding’ stages of *Dermanyssus gallinae*. The transcripts localising to the individual life stages (egg, larvae, protonymph, deutonymph, adult female and adult male) and multiple blood feeding life stages (protonymph, deutonymph, adult female and adult male) of the Venn/Eular diagram were organized in broad categories indicative of their biological function (Gene function category and Inferred function) based on the comprehensive assessment of data from Blastp homology, associated GO annotations and InterPro terms.

**Additional file 5: Supplementary file 5.** Differentially expressed genes (DEGs) at a simulation probability of *P* > 0.95. Fifteen pairwise comparisons were performed at the simulation probability cut-off of P > 0.95 between the different *Dermanyssus gallinae* life stages as shown.

**Additional file 6: Supplementary file 6.** Differentially expressed genes (DEGs) at a simulation probability of *P* > 0.99. Fifteen pairwise comparisons were performed at the simulation probability cut-off of *P* > 0.99 between the different *Dermanyssus gallinae* life stages as shown.

**Additional file 7: Supplementary file 7.** Putative *Dermanyssus gallinae* homologues of the 39 house dust mite allergens classified by the WHO/International Union of Immunological Societies Allergen Nomenclature Subcommittee (WHO/IUISAN http://www.allergen.org/).

## Data Availability

The datasets generated and analysed during the current study are fully compliant with the MINISEQE guidelines and are deposited in the publicly accessible NCBI Sequence Read Archive (SRA) Database under the project accession number PRJNA682816. The *D. gallinae* draft genome sequence is available at DDBJ/ENA/GenBank under the accession number QVRM00000000. The full annotation of the *D. gallinae* genome has been made publicly available via the Online Resource for Community Annotation of Eukaryotes (OrcAE) via the following link: https://bioinformatics.psb.ugent.be/orcae/
